# Self-(In)compatibility Systems: Target Traits for Crop-Production, Plant Breeding, and Biotechnology

**DOI:** 10.3389/fpls.2020.00195

**Published:** 2020-03-19

**Authors:** Juan Vicente Muñoz-Sanz, Elena Zuriaga, Felipe Cruz-García, Bruce McClure, Carlos Romero

**Affiliations:** ^1^Department of Biochemistry, University of Missouri, Columbia, MO, United States; ^2^Centro de Citricultura y Producción Vegetal, Instituto Valenciano de Investigaciones Agrarias (IVIA), Valencia, Spain; ^3^Departmento de Bioquímica, Facultad de Química, Universidad Nacional Autonoma de Mexico, Mexico City, Mexico; ^4^Instituto de Biología Molecular y Celular de Plantas (IBMCP), Consejo Superior de Investigaciones Científicas (CSIC)—Universitat Politécnica de València (UPV), Valencia, Spain

**Keywords:** self-(in)compatibility, *S*-genotyping, interspecific reproductive barriers, hybrid breeding, crop production, plant breeding

## Abstract

Self-incompatibility (SI) mechanisms prevent self-fertilization in flowering plants based on specific discrimination between self- and non-self pollen. Since this trait promotes outcrossing and avoids inbreeding it is a widespread mechanism of controlling sexual plant reproduction. Growers and breeders have effectively exploited SI as a tool for manipulating domesticated crops for thousands of years. However, only within the past thirty years have studies begun to elucidate the underlying molecular features of SI. The specific *S*-determinants and some modifier factors controlling SI have been identified in the sporophytic system exhibited by *Brassica* species and in the two very distinct gametophytic systems present in Papaveraceae on one side and in Solanaceae, Rosaceae, and Plantaginaceae on the other. Molecular level studies have enabled SI to SC transitions (and *vice versa*) to be intentionally manipulated using marker assisted breeding and targeted approaches based on transgene integration, silencing, and more recently CRISPR knock-out of SI-related factors. These scientific advances have, in turn, provided a solid basis to implement new crop production and plant breeding practices. Applications of self-(in)compatibility include widely differing objectives such as crop yield and quality improvement, marker-assisted breeding through SI genotyping, and development of hybrids for overcoming intra- and interspecific reproductive barriers. Here, we review scientific progress as well as patented applications of SI, and also highlight future prospects including further elucidation of SI systems, deepening our understanding of SI-environment relationships, and new perspectives on plant self/non-self recognition.

## Introduction

### Early Background on Self-Incompatibility

Humans have been aware of the link between pollination and seed production since the Neolithic period, as reflected by the laws of Hammurabi (1750 B.C.E.), these being integral parts of agriculture development (Weiss, 2015). This basic knowledge is represented in several Assyrian reliefs dating to the reign of Ashurnasirpal II (884–859 B.C.E.) that show gods and priests performing fertilization rituals of date palms (Weiss, 2015). While people have been benefitting from sexual reproduction for thousands of years, the male and female plant reproductive organs were not explicitly documented until the publication of Rudolf Jakob Camerarius’s “De sexu *Plantarum* epistole” in 1694 (Abrol, 2012). In 1764, Joseph Gottlieb Kölreuter reported the occurrence of self-sterility and performed cross-pollinations to obtain interspecific hybrids in *Verbascum* (East and Park, 1917). A century later, Charles Darwin established the influence of self-incompatibility (SI) in plants in his works, *The effects of cross and self-fertilization in the vegetable kingdom* (1876) and *The different forms of flowers on plants of the same species* (1877) (McClure, 2009).

Botanists subsequently demonstrated, in *Reseda* and *Nicotiana*, that self-sterility follows Mendelian inheritance (East and Park, 1917). The *S*-locus and its multi-allelic nature were then shown to genetically control self-sterility in *Nicotiana*, leading to intrasterile and interfertile compatibility classes (East and Yarnell, 1929). In light of these findings, the term “self-sterility” was progressively replaced with the term “self-incompatibility” (McClure, 2009). By then, breeders from both public institutions and private companies focused interest on SI as a tool. The John Innes Horticultural Institution has been studying incompatibility and sterility in plums, cherries, and apples since 1911, and in pears since the 1930s (Crane and Lewis, 1942). For instance, cross-pollinations were used to define intercompatible groups in sweet cherry (*Prunus avium* L.) cultivars (Crane and Brown, 1937), and afterwards a pollen irradiation program produced the first self-compatible (SC) cultivars within this strictly SI species (Lewis and Crowe, 1954). In 1940, Sakata Seed Company introduced the F_1_-hybrid cabbage cv. Suteni Kanran, produced using SI. This success was soon followed by Takii & Co. Ltd’s introduction of the cabbage (*Brassica oleracea* L.) cvs. Choko-1c and Choko-1cc in 1950 (Watanabe et al., 2008).

Early commercial interest in SI was not restricted to fruit trees (Rosaceae) and cabbages (Brassicaceae). It extended to other crop species, including potato (*Solanum tuberosum* L.), sunflower (*Helianthus annuus* L.), rye (*Secale cereale* [L.] M. Bieb.), cocoa (*Theobroma cacao* L.), and pummelo (*Citrus grandis* Osbeck) [see De Nettancourt (2001) for a full review of the early works on SI in a broad range of species].

The underlying molecular basis for SI remained a black box until the mid-1980s. Subsequent discoveries have generated new avenues for manipulating SI to the benefit of crop production and plant breeding. To date, consistent evidence identifying the molecular determinants of SI is available in Brassicaceae, Rosaceae, Solanaceae, Plantaginaceae, Rubiaceae, and Papaveraceae, although a plethora of studies are underway in additional species.

### Molecular Mechanisms of SI

Recent reviews of SI mechanisms provide detailed descriptions of molecular and genetic mechanisms (McClure et al., 2011; Iwano and Takayama, 2012; Wilkins et al., 2014; Fujii et al., 2016; Bedinger et al., 2017; Sehgal and Singh, 2018; Wang et al., 2018). Here, we provide an overview sufficient for discussing aspects relevant to crop breeding and production. SI prevents self-fertilization based on the discrimination between self- and non-self pollen. It has been reported in more than 100 plant families and occurs in approximately 40% of species (Igic et al., 2008) including many important crops (e.g., canola, potato, pome and stone fruits, olive, cocoa, tea, coffee, etc.) and/or their wild relatives. In many angiosperms, SI is genetically controlled by a single multiallelic locus, termed the *S*-locus, though systems controlled by two (or more) loci have also been described in certain species (e.g. grasses) (De Nettancourt, 2001). The *S*-locus encodes both male and female specificity determinants (*S*-determinants) whose products are predicted to interact and trigger the self/non-self discrimination process (Iwano and Takayama, 2012). Most types of SI can be classified as sporophytic or gametophytic based on time of gene action in the stamen (De Nettancourt, 2001). The pollen phenotype is determined by the *S*-genotype of the diploid pollen-parent in sporophytic SI and by the genotype of the individual microspore in gametophytic SI. Three SI mechanisms have been characterized at the molecular level. Sporophytic self-incompatibility (SSI) has been elucidated in Brassicaceae, and two distinct types of gametophytic self-incompatibility (GSI) have been extensively studied, S-RNase-based SI in Solanaceae and Rosaceae, and the *Papaver* system based on programmed cell death (PCD).

In Brassicaceae, *S*-locus genes encode serine/threonine receptor kinase (SRK) (Takasaki et al., 2000) and cysteine-rich (SP11/SCR) proteins (Schopfer et al., 1999) that, respectively, form the female and male *S*-determinants. Both *S*-locus genes, and a third gene encoding a glycoprotein (*SLG*) that may enhance SI expression (Takasaki et al., 2000), are tightly linked and inherited as an *S*-haplotype (Sehgal and Singh, 2018). SRK protein is localized to the plasma membrane of the papilla stigmatic cells, and the small SP11 polypeptide is secreted from the anther tapetum, deposited onto the pollen coat, and finally acts as an SRK ligand upon pollination (Iwano and Takayama, 2012). Many Brassicaceae *S*-haplotypes have been identified. They exhibit complex hierarchical dominance relationships that are controlled by polymorphic small RNAs and their targets (Yasuda et al., 2016). It should be noted that these dominance relationships complicate the observed compatibility patterns and are, thus, of considerable practical importance. However, in uncomplicated examples, specific interaction between SP11 and SRK from the same *S*-haplotype triggers the self-pollen rejection mechanism in the stigma papillar cell. Downstream events are controlled by additional factors. The M-locus protein kinase (MLPK) interacts with SRK to transduce SI signaling and an arm repeat containing 1 U-box type E3-ligase (ARC1) ubiquitinates and degrades the Exo70A1 factor required for pollen growth. On the contrary, other factors such as the thioredoxin h-like 1 (THL1) and the kinase-associated protein phosphatase (KAPP) inhibit SRK and act as negative regulators of the SI response (Cabrillac et al., 2001). The interaction between SRK and SP11 has also been shown to lead to an increase in cytosolic [Ca^2+^] in the papilla cell but how this calcium influx prevents self-pollen growth is unclear (Iwano et al., 2015).

S-RNase based GSI is present in widely divergent families (i.e., Rosaceae, Solanaceae, Scrophulariaceae, Rubiaceae). Surprisingly, although different taxa use similar genes to determine the specificity of pollen rejection, the detailed mechanisms show important differences. Nevertheless, in across all families, the *S*-locus contains at least two linked genes (although often many more). In every case, one gene encodes pistil-expressed glycoproteins with ribonuclease activity (S-RNases) that act as highly selective cytotoxins that cause rejection of pollen when its single *S*-haplotype matches either of the two *S*-haplotypes in the diploid pistil (McClure et al., 1989; Boskovic and Tobutt, 1996; Xue et al., 1996). The other is an F-box protein gene specifically expressed in pollen, termed *SLF* or *SFB*, depending on the family. Importantly, *S*-haplotypes in Solanaceae and in the Rosaceae tribe Maleae (apple, pear) have an array of 16 to 20 *SLF* genes that collectively contribute to pollen SI functions (Kubo et al., 2010; Kakui et al., 2011; Williams et al., 2014). In contrast pollen-side function in SI *Prunus* species (Rosaceae) is provided by a single *SFB* gene (Ushijima et al., 2004; Sonneveld et al., 2005). The F-box protein gene was first identified in *Antirrhinum* (Lai et al., 2002) and later in *Prunus* (Entani et al., 2003; Ushijima et al., 2003) and *Petunia* (Sijacic et al., 2004). F-box proteins are best known for their roles in the 26S ubiquitin/proteasome pathway (by forming the SCF complex along with SKP1 and Cullin1 proteins) and the reported interaction between the AhSLF_2_ F-box protein and self/cross S-RNases in *Antirrhinum* pollen suggested that cross S-RNases might be inactivated through this pathway (Qiao et al., 2004). These and other findings support a model where non-self S-RNases are degraded in compatible pollinations, but in self-pollinations self S-RNases evade degradation and degrade the pollen RNA. In Solanaceae, ubiquitation and degradation of S-RNase is attributed to the collective action of the array of 16 to 20 SLF proteins (Kubo et al., 2010), but self-S-RNase is not degraded because it fails to be recognized (Kubo et al., 2015). This is referred to as the collaborative non-self recognition model (i.e., the array of SLF proteins recognizes non-self S-RNase) and it is currently the most widely accepted model. However, S-RNases are also sequestered in the pollen tube endomembrane system and this may also contribute to compatibility (Goldraij et al., 2006). Remarkably, knock-out mutations in *Prunus SFB* genes confer SC at odds with the collaborative non-self recognition model prediction. Thus, an alternative model has been suggested in *Prunus* where self-SFB protects self-S-RNases from a “general inhibitor” (proposed to be the *S*-locus linked SLF-like2 factor) that detoxifies all self/nonself-S-RNases (Matsumoto and Tao, 2016). Also, the collaborative non-self recognition model only addresses the roles of S-RNase and SLF proteins and there is compelling evidence that pistil factors (modifiers) that do not contribute to *S*-specificity are nevertheless required for SI in Solanaceae, including HT-B (McClure et al., 1999), 120 kDa glycoprotein (Hancock et al., 2005), the thioredoxin NaTrxh (Juárez-Díaz et al., 2006), the proteinase inhibitor NaStEP (Busot et al., 2008), and the mitochondrial phosphate carrier NaSIPP (García-Valencia et al., 2017). Moreover, pollen modifiers have been identified in Rosaceae including an ATP-Binding Cassette F-protein (ABCF) transporter in *Malus* (Meng et al., 2014) as well as the *Prunus armeniaca* M-locus disulfide bond A-like oxidoreductase (ParMDO) (Muñoz-Sanz et al., 2017a) and the *P. avium* M-locus glutathione S-transferase (MGST) (Ono et al., 2018). Clearly, although enormous progress has been made, much remains to be learned about S-RNase-based SI.

The physiology of GSI in poppy (*Papaver rhoeas* L.) has been elucidated more fully than any other system. The *S*-locus comprises two tightly linked genes that encode the female (PrsS) and male (PrpS) *S*-determinants. PrsS is a small, highly polymorphic protein secreted by stigmatic papilla cells (Foote et al., 1994) that acts as a signaling ligand to interact with the pollen-expressed transmembrane protein PrpS (Wheeler et al., 2009).The self-interaction triggers a range of responses, including an increase in cytosolic free Ca^2+^, an influx of Ca^2+^ and K^+^, and the production of reactive oxygen species (ROS) and nitric oxide. These processes, in turn, act on downstream targets. The soluble organic pyrophosphatase p26 and the MAP Kinase p56 are rapidly phosphorylated while the actin cytoskeleton is progressively depolymerized leading to PCD. DNA fragmentation and a caspase-like activity, other hallmarks of PCD, are also detected (Wilkins et al., 2014). SI-induced acidification of the pollen tube cytosol has also recently been identified as a trigger for PCD. Reduced cytosolic pH signals to the SI-induced caspase3-like activity, reduction of the p26 pyrophosphatase activity, formation of filamentous actin (F-actin) foci, and their colocalization with certain actin binding proteins, but further exploration is still warrented (Wang et al., 2018).

## Applications to Crop Production and Breeding

Our increasing understanding of the molecular basis of SI is providing new opportunities to exploit this trait for crop improvement by breeding and biotechnology-based approaches. Applications derived from current knowledge on SI are diverse but can be grouped into three main categories: crop production (yield and quality), marker-assisted breeding, and hybrid development (intra- and interspecific).

### SI Versus SC in Crop Production, Yield, and Quality

#### Pollenizers and Orchard Management

In crops exhibiting SI (and even in SC crops), cultivars that serve as pollen donors (“pollenizers”) are usually interspersed throughout orchards since fruit set depends largely on cross-pollinations. Pollenizers are commonly used in canola (*Brassica napus* L.), sunflower, strawberry (*Fragaria* x *anannasa* [Weston] Duchesne), and fruit trees such as apple (*Malus x domestica* L.), European pear (*Pyrus communis*), sweet cherry, Japanese plum (*Prunus salicina* Lindl), etc. (Woodcock, 2012). The use of pollenizers is also recommended in olive (*Olea europaea* L.) where the recent discovery of homomorphic sporophytic diallelic SI (DSI) should facilitate their selection (Saumitou-Laprade et al., 2017).

In diploid fruit tree species that display GSI, outcrosses are classified into three types: incompatible (parents share both *S*-haplotypes), semi-compatible (one shared *S*-haplotype), and fully compatible (no shared *S*-haplotype). In semi-compatible crosses, half the available pollen grains are rejected, which may have a significant impact on fruit set and yield (fruit size), for instance, in apple, European pears, and Japanese plums when grown in sub-optimal regions (e.g., the Mediterranean basin) (Schneider et al., 2005; Zisovich et al., 2005; Sapir et al., 2008). When honeybee visits are boosted, semi-compatible cultivars show increased yields, confirming that lack of compatible pollen is responsible for yield reduction (Stern et al., 2001; Stern et al., 2004; Sapir et al., 2007). In addition, in genera with many ovules such as *Malus* and *Pyrus*, a reduction in fertilization may result in fewer seeds and inferior fruit quality. In these and other species, SC is linked to satisfactory fruit set, superior yields, or even over-cropping (Goldway et al., 2007; Claessen et al., 2019). SC may also be desirable because it obviates the need for pollenizers (relying on bloom overlapping) and, thus, allows a single cultivar to be grown in a “solid block” to produce a more uniform crop. SC also has been considered to offset the effects of colony collapse disorder on honeybee pollination (e.g., in the California almond industry). However, while SC may reduce the number of hives required, it cannot always guarantee full yields, as some crops require cross-pollination for maximum fruit set (e.g., sunflower, canola, sour cherry [*Prunus cerasus* L.], almond [*Prunus dulcis* {Mill.} D.A.Webb.], apricot [*Prunus armeniaca* L.], etc.). For instance, some SC apple and pear varieties, where SC may be associated with defective expression of the *S-RNase*, show low fruit set, but fruit set is restored (and displaces self-pollination) if cross-pollen is available (Schneider et al., 2001; Zhang and Hiratsuka, 2005). In addition, SC is not always sufficient for self-fruitfulness since insects may be needed for effective self-pollination because of the flower structure. Conversely, honeybee pollination may induce over-pollination in SC stone fruits (i.e., sour cherry, apricot, peach [*Prunus persica* {L.} Batsch]) leading to overly heavy fruit set and undersized fruits of reduced value (Woodcock, 2012). Also, this may eventually occur with SI cultivars. Thus, fruit set is not only SI/SC phenotype dependent but also cultivar dependent.

#### Different Sources of SC: A Favorable Trait for Yield Enhancement

**Figure 1:**
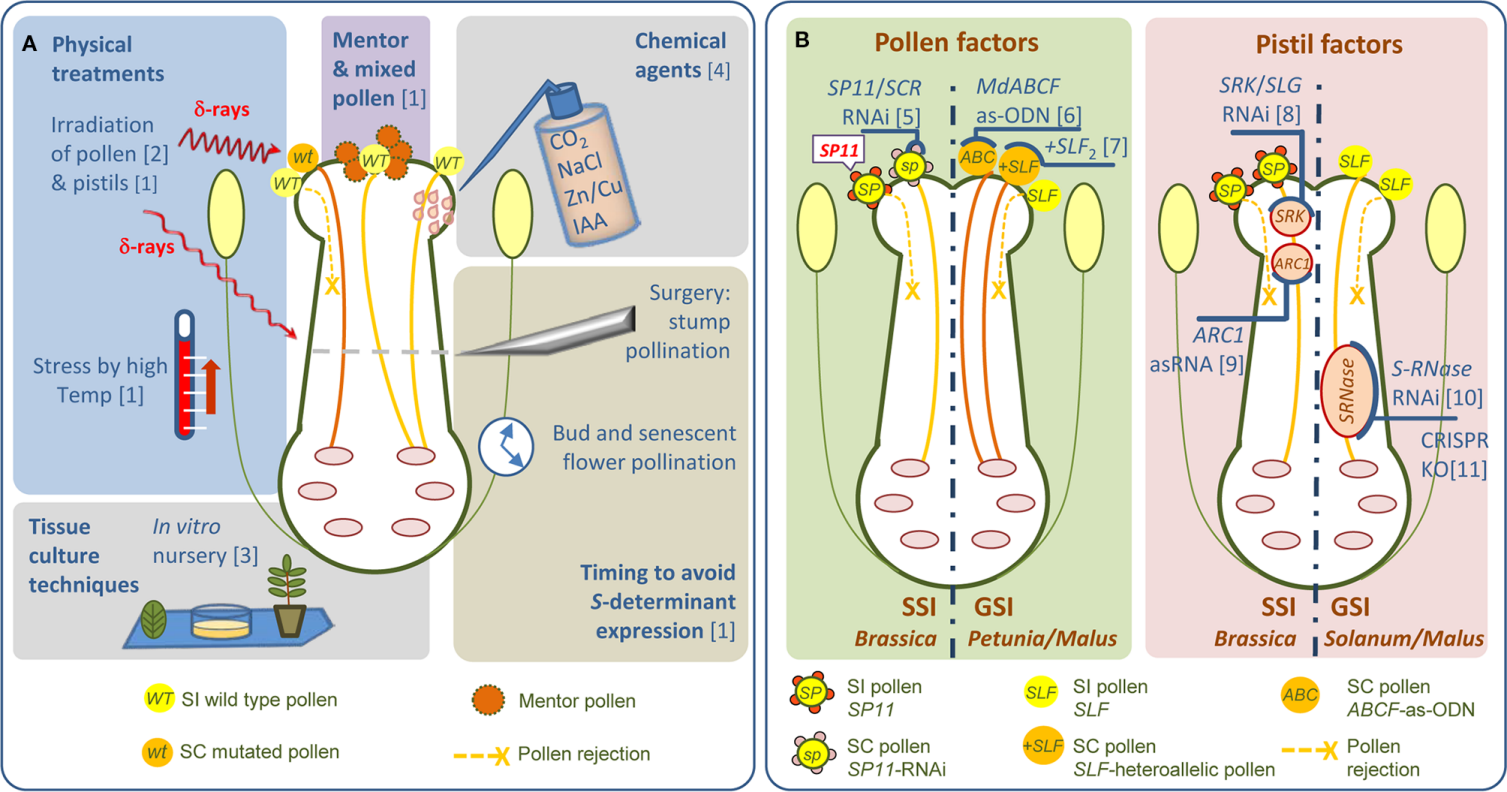
Untargeted and molecular-targeted strategies for overcoming SI. **(A)** The most common types of untargeted strategies to overcome SI are shown. **(B)** Molecular-based strategies in crop species exhibiting the SSI and GSI systems. Strategies to down-regulate pollen- and pistil-expressed genes by RNAi/as-ODN/as-RNA or CRISPR. RNAi, RNA interference; as-ODN, antisense-oligodeoxynucleotide; asRNA, antisense RNA. References: [1] De Nettancourt (2001); [2] Lewis and Crowe (1954); [3] De La Fuente et al. (2013); [4] Kučera et al. (2006); [5] Jung et al. (2012); [6] Meng et al. (2014); [7] Sijacic et al. (2004); [8] Shiba et al. (1995); [9] Stone et al. (1999); [10] Broothaerts et al. (2004); [11] Ye et al. (2018).

In otherwise SI crops, most commercial SC cultivars derive from spontaneous and induced style- or pollen-part mutations identified and selected by growers and breeders (**Figure 1A**). These mutations are continually being characterized at the molecular level but use of uncharacterized sources of SC remains common. In stone fruits (*Prunus*), such as apricot, Japanese apricot (*Prunus mume* Sieb. Et Zucc.), Japanese plum, or almond, SC is usually linked to a particular self-fertility *S*-locus allele (i.e., *S*_C_, *S*^f^, *S*_e_, and *S*_f_, respectively) (Yamane and Tao, 2009). *Prunus* SC alleles with loss-of-function *S*-*RNase* mutations are known, but *SFB* mutations are more common (Yamane and Tao, 2009). For example, in the 1940s the John Innes Center sweet cherry breeding program used pollen x-ray irradiation to induce SC in ‘Napoleon.’ A number of SC commercial cultivars derived from ‘Napoleon,’ including ‘Stella,’ ‘Lapins,’ ‘Newstar,’ and ‘Sweethart’ were thus obtained. Much later, all these SC cultivars were shown to possess a common defective (mutated) *SFB*^4´^ allele (Ushijima et al., 2004; Sonneveld et al., 2005). In apricot cultivars, SC is mainly conferred by the mutant *SFB*_C_ allele, but an unlinked mutation at the M-locus *ParMDO* gene represents another source of SC in varieties such as ‘Canino,’ ‘Patterson,’ ‘Trevatt,’ and ‘Portici’ (Muñoz-Sanz et al., 2017a; Muñoz-Sanz et al., 2017b). Similarly a mutation at the M-locus *GST* gene confers SC in the sweet cherry cultivar ‘Cristobalina’ (Wunsch and Hormaza, 2004; Ono et al., 2018). In another example, SC turnip (*Brassica rapa* L.) cultivars ‘Yellow Sarson’ and ‘Dahuangyoucai’ carry indels affecting *SRK* and *SP11* as well as a knockout point mutation in the non-*S*-locus gene *MLPK* (Zhang et al., 2013).

Knowledge of the molecular and genetic basis of SI has enabled SC to be engineered in a targeted manner (**Figure 1B**). Shiba et al. (1995) were the first to successfully use a targeted strategy to overcome SI. They obtained SC *Brassica rapa* by suppressing *SLG* expression, and most likely *SRK* expression as well due to their high sequence similarity, using an antisense *SLG* construct. Using a similar approach, Stone et al. (1999) produced SC to the otherwise SI *Brassica napus* cv. W1 by introducing an antisense *ARC1* cDNA. Much later, Jung et al. (2012) developed SC *B. rapa* using RNAi-mediated *S*-locus gene silencing. This RNAi construct was prepared from the *S*_60_-allele of the pollen *S*-determinant, SP11/SCR (cv. ‘Osome’). Transgenic RNAi lines were shown to be stable and highly SC (**Figure 1B**). Transfer of this engineered SC into the commercial SI variety ‘Seoulbechhu’ demonstrates the utility of this approach for breeding. In pome fruits Broothaerts et al. (2004) developed a self-fertile apple cultivar (US Patent No 20060123514) by silencing *S-RNase* gene expression in SI cv. ‘Elstar’ (*S*_3_*S*_5_ genotype) (**Figure 1B**). They described two independent transgenic trees expressing an *S*_3_-*RNase* antisense construct. These trees showed normal pollen tube growth and fertilization after selfing and produced normal levels of fruits and seeds. Interestingly, both *S*_3_- and *S*_5_-*RNases* were silenced, presumably because of sequence similarity. SC was retained in these trees over several years without obvious adverse effects. Much more recently, CRISPR-induced knockouts of *S-RNase* have been used to generate SC diploid lines for breeding purposes in potato (*Solanum tuberosum* L.) (Ye et al., 2018; Enciso-Rodriguez et al., 2019) (**Figure 1B**).

#### Instances Where SI is Preferred for Crop Production

When the absence of seed or fertilization is desirable SI may be the preferred condition. For instance, seedless fruits are highly desirable in some crops such as citrus (e.g., orange, mandarin, lemon, etc.). Most citrus cultivars display some degree of parthenocarpy and, thus, they form normal, but seedless, fruits without fertilization. However, when otherwise seedless cultivars are cultivated in proximity of cross-compatible cultivars undesirable seeded fruits may still be formed. Consequently, SI is regarded as a target trait for *Citrus* breeding so that it could be used in conjunction with parthenocarpy to greatly reduce seed number (Vardi et al., 2008). This approach was validated with a mutant mandarin (*Citrus reticulata* Blanco) cv. ‘Wuzishatangju’ where seedlessness could be attributed to GSI (Ye et al., 2009). Furthermore, roles for GSI in seedlessness also have been reported in the mandarin cv. ‘Afourer’ (Gambetta et al., 2013) and in the lemon (*Citrus limon* [L.] Burm. F.) cultivars ‘Xiangshui’ (Zhang et al., 2012) and ‘Kagzi kalan’ (Kakade et al., 2017). Style-expressed *S*-like-RNases and *SKP*1-like genes have been proposed to be involved in the SI response of the mandarin cv. ‘Wuzishatangju’ (Miao et al., 2011; Li et al., 2015). However, more research on the *Citrus* SI system will be needed before engineering SI for seedlessness will be practical.

### Molecular *S*-Genotyping for Successful Crossing and Marker-Assisted Breeding

Specific knowledge of the level of cross-compatibility between cultivars is important to the seed and fruit industries. In species where the *S*-determinants have been identified, molecular genotyping has progressively replaced controlled pollinations, pollen tube growth tests, and enzymatic assays used to determine the *S*-genotype. Molecular genotyping also has accelerated the identification of new *S*-alleles, because it does not depend on environmental conditions and, in the case of trees, it does not require adult plants (Yamane and Tao, 2009). This has been especially useful for producers wishing to select efficient pollenizers (see *Pollenizers and Orchard Management*). For example, Sapir et al. (2008) reported that 87% of the Japanese plum germplasm grown in Israel contained only four *S*-haplotypes and this allowed them to focus their search for fully compatible cultivars. This is not an isolated example. Over the past 25 years, molecular *S*-genotyping has doubled the number of identified intercompatibility groups in most fruit tree species (Yamane and Tao, 2009). *S*-genotyping also has been used to design crosses and to select SC hybrids produced in breeding programs where, in many cases, SI parents are still needed.

In species with S-RNase-based SI, *S*-genotyping is usually based on the variability in intron sizes among the different *S*-*RNase* alleles. It is noteworthy that *Prunus* spp., *S*-*RNase* genes contain two introns while all other species have a single *S-RNase* intron (Tao et al., 1999; Igic and Kohn, 2001). Often, conserved or specific primers flanking *S*-*RNase* introns are used to amplify PCR different sized products that are *S*-genotype specific. This approach has been extensively used for *S*-genotyping in many SI Rosaceae crop species, including pome and stone fruits (**Table 1**). High-throughput methods have also been developed for *S-RNase*-based *S*-genotyping. For instance, microarray platforms based on intron and cDNA sequences have been developed to identify *S*-*RNase* alleles in sweet cherry (Pasquer et al., 2008) and Asian pear (*Pyrus pyrifolia* Nakai) (Nan et al., 2015), respectively. In apple, Larsen et al. (2016) identified 25 *S*-*RNase* alleles in 432 accessions by using multiplex PCR and fragment detection on a capillary DNA sequencer (**Table 1**).

**Table 1:** Molecular *S*-genotyping in crop species.

Species	*S*-alleles/*S*-haplotypes^a^	Method	*S*-genes	*N*^b^	Refs.
**Brassicaceae**					
Cabbage, broccoli	16 (S^a^-S^p^)	PCR-RFLP ^c^	*SLG*	40	Park et al. (2001)
Cabbage, cauliflower	17 (Bo-Bob)	PCR-RFLP	*SLG/SRK*	30	Wang et al. (2007)
Cabbage	40 (*BrS*-)	Dot-blot	*SP11*	45	Oikawa et al. (2011)
Cabbage	16 (S)	Sequencing	*SRK/SP11*	107	Tian et al. (2013)
(*Brassica oleracea*)					
Turnip	33 (*BoS*-)	Dot-blot	*SP11*	42	Oikawa et al. (2011)
Chinese cabbage, etc.	26 (Bc-Bcp)	PCR-RFLP	*SLG/SRK*	38	Wang et al. (2007)
(*Brassica rapa*)					
Mustard	1 (Bj)	PCR-RFLP	*SLG/SRK*	4	Wang et al. (2007)
(*Brassica juncea*)					
Oilseed rape (*Brassica napus*)	4/2 (*S-I*and*S-II_SLG_^a,b^*)/(*S-II_SP11_^a,b^*)	PCR-CAPS	*SLG/SP11*	125	Zhang X. et al. (2008)
Radish	18 (*S*^1^-*S*^18^)	Southern	*SLG*	29	Sakamoto et al. (1998)
(*Raphanus sativus*)	7/7 (S_1-10_/S_1-10)_)^a^	PCR-RFLP	*SLG/SRK*	24	Lim et al. (2002)
	9/10 (Rs-SRK_1–21_/-SP11_1-21_)^a^	Sequencing	*SRK/SP11*	10	Okamoto et al. (2004)
	15 (RsS_1-40)_)^a^	Sequencing	*SLG/SRK*	63	Wang et al. (2019)
**Rosaceae (tribe Amygdaleae)**					
Apricot (*Prunus armeniaca*)	30 (S_1_-S_20_, S_22_-S_30_, S_C_)	PCR/Sequencing	*S-RNase/SFB*	261	Halázs et al. (2007a); Halázs et al. (2010a); Zhang L. et al. (2008); Wu et al. (2009); Lachkar et al. (2013); Muñoz-Sanz et al. (2017b); Herrera et al. (2018)
Japanese apricot (*Prunus mume*)	13 (S^1^-S^11^, S^f^, S^3´^)	PCR	*S-RNase*	16	Yamane and Tao (2009)
Japanese plum (*Prunus salicina*)	19 (S^a^-S^s^)	PCR	*SRNase/SFB*	149	Beppu et al. (2002); Beppu et al. (2003); Sapir et al. (2004); Zhang et al. (2007); Halázs et al. (2007b); Guerra et al. (2009); Guerra et al. (2012)
European plum (*Prunus domestica*)	18 (S_A_-S_S_)	PCR	*S-RNase*	16	Halázs et al. (2014)
Peach	3 (S^1^, S^2^, S^2m^, S^3^, S^4^)	PCR	*S-RNase*	195	Hegedüs et al. (2006); Tao et al. (2007); Hanada et al. (2014)
(*Prunus persica*)		PCR-CAPS			
Almond (*Prunus dulcis*)	34 (S_1_-S_52_, S_f_)^a^	PCR/Sequencing	*S-RNase*	170	López et al. (2006); Ortega et al. (2006); Kodad et al. (2008); Halazs et al. (2010b); Currò et al. (2015)
Sweet cherry (*Prunus avium*)	18 (S^1^-S^24^)^a^	PCR-RFLP PCR/Sequencing Microarray	*S-RNase/SFB*	≳700	Wiersma et al. (2001); Wunsch and Hormaza (2004); Vaughan et al. (2006); Schuster (2012); Cachi and Wünsch (2014); Pasquer et al. (2008)
Sour cherry (*Prunus cerasus*)	15 (S_1-36_, S_1´_, S_13´_, S_6m-_S_36a-b-b2_)^a^	PCR	*S-RNase*	21	Lisek et al. (2017)
**Rosaceae (tribe Maleae)**					
Apple (*Malus x domestica*) Crabapple (*Malus* spp.)	31 (S^1^-S^46^)^a^	PCR-CAPS/PCR Mplex PCR PCR-RFLP	*S-RNase*	596	Yamane and Tao (2009); Kim H. T. et al. (2009); Long et al. (2010); Larsen et al. (2016); Sheick et al. (2018)
European pear (*Pyrus communis*)	21 (S_1_-S_24;_ Sm)^a^	PCR/Sequencing	*S-RNase*	201	Sanzol and Robbins (2008); Goldway et al. (2009); Sanzol (2009); Quinet et al. (2014)
Japanese pear (*Pyrus pyrifolia*)	39(S^1^-S^52^, S^4sm^, S^k^)^a^	PCR-RFLP/PCR Microarray	*S-RNase*	101	Yamane and Tao (2009); Gu et al. (2009); Nashima et al. (2015); Nan et al. (2015)
Loquat (*Eriobotrya japonica*)	13 (S_a_-S_k_, S^12^-S^13^)	PCR	*S-RNase*	150	Gisbert et al. (2009); Carrera et al. (2011); Wang et al. (2017); Zhang et al. (2017)
**Rubiaceae**					
Coffee (*Coffea* spp.)	14 species including *C. arabica*. 36 (S_1_-_51_)^a^	Sequencing	*S-RNase*	58	Nowak et al. (2011)
**Solanaceae**					
Tomato (*Solanum subsection Lycopersicon*)	5 species including *S. arcanum, S. hirsutum*, etc. 24 (S_6_-S_26_, S_C_)^a^, (S_1_-S_2_), etc. *S. chilense* 30 (S_1_-S_30_)	Sequencing Sequencing	*S-RNase* *S-RNase*	21 34	Kondo et al. (2002) Igic et al. (2007)
Potato (*Solanum* subsection *Petota*.)	5 species including *S. chacoense, S. S. stenotonum*, etc. 25 (S_2_-S_3_, S_11_-S_16_), (Ss_1_-Ss_10_), etc.	Sequencing	*S-RNase*	14	Dzidzienyo et al. (2016)

a Numbered non-consecutively.

b N Number of cultivars or accessions analyzed.

c Restriction Fragment Length Polymorphisms.

*S*-genotyping is applicable when a single gene determines phenotype. This is the case for *S-RNase*, but also applies to other genes. In *Prunus*, a single *SFB* gene specifies pollen-part behaviour so these genes also can be used for molecular *S*-genotyping. Vaughan et al. (2006) identified an intron in the sweet cherry *SFB* 5´untranslated region that shows allele-specific length polymorphism and used an automatic sequencer and fluorescent primers for *S*-genotyping. This method has also been widely applied in Japanese plum (Guerra et al., 2012) and apricot (Muñoz-Sanz et al., 2017b). In a related approach, that is independent of the *S*-locus *per se*, apricot self-(in)compatibility phenotypes were assessed by genotyping the SI modifier gene *ParMDO* at the M-locus (Muñoz-Sanz et al., 2017b). In Brassicaceae, extensive *S*-haplotyping in *B. rapa* and *B. oleracea* has been performed by dot-blot analysis with digoxigenin-labeled PCR products amplified from *SP11* DNA sequences (Oikawa et al., 2011).

*S*-genotyping also may be applied to wild crop relatives. For example, cultivated tomato, potato, and coffee (*Coffea arabica*) are SC, but wild relatives carrying valuable pest and disease resistance traits are mostly SI (Dzidzienyo et al., 2016). Cultivated and wild diploid potatoes, however, are usually SI, so crosses to create pre-breeding materials can be facilitated by knowledge of their *S*-genotypes (**Table 1**). Similarly, coffee cultivars are SC tetraploids while its parent, *C. canephora* (robusta coffee), is diploid and mostly SI. Although SC *C. arabica* is more widely cultivated, *C. canephora* is also economically important and shows higher fitness, as do other wild relatives (Asquini et al., 2011). Thus, *S*-genotyping has been applied to wild *Coffea* species including *C. canephora* (Nowak et al., 2011). Selection of pollinizers based on *S*-genotyping is also relevant in this context since wild relatives are sometimes used. For example, wild crabapples (*Malus* spp.) may be used in commercial apple orchards to promote cross-pollination (Sheick et al., 2018).

*S*-genotyping also provides “value-added” markers for marker-assisted breeding. Over the last decade, SSRs and SNPs have become the “markers of choice” for genotyping. While SNPs are more numerous than SSRs, the latter have distinct advantages (Guichoux et al., 2011). One such benefit is the high mutation rate and multi-allelic nature of SSRs compared with SNPs, and this feature is shared by the *S*-locus. The extremely polymorphic nature of the *S*-locus is related to the frequency-dependent selection on *S*-haplotypes inherent in SI: by definition, plants with rare *S*-haplotypes have more compatible mates than those with common *S*-haplotypes (Schueler et al., 2006). *S*-locus polymorphism can be applied in several ways. Although *S*-genotyping only provides information about a short stretch of the genome, breeding programs nevertheless use it to detect undesired crosses (e.g., accidental pollinations, or self-fertilizations arising when SC females are used), or seed resulting from asexual reproduction. Molecular *S*-genotyping is also indicated when SI phenotyping is difficult and expensive, as with fruit trees. For instance, a DNA test based on the *S*_4_’-allele conferring SC in cherry, together with another test for fruit size, is now routinely used in a streamlined marker-assisted seedling selection scheme by a Pacific Northwest sweet cherry breeding program (Ru et al., 2015) and it is most likely used in all sweet cherry breeding programs implementing MAS. In another example, gene-specific *S*-locus markers have been developed at the Saskatoon Research and Development Center in Canada to select SC genotypes in yellow mustard (*Sinapis alba* L.) (Zeng and Cheng, 2014).

### SI as an Alternative to Androsterility for Developing Hybrids

**Figure 2:**
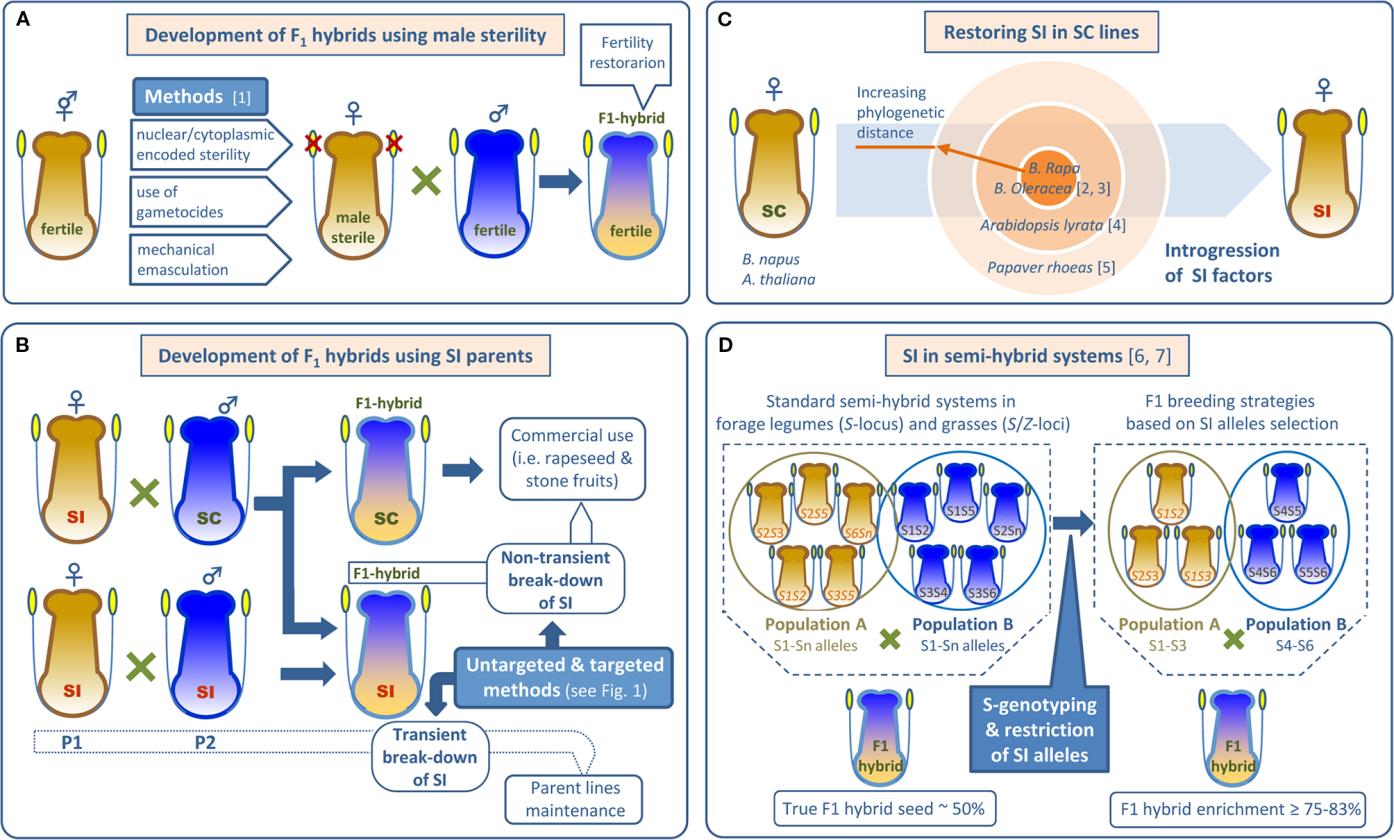
Use of male-sterility and self-incompatibility in F_1_ hybrid seed production. **(A)** Common methods to prevent self-fertilization of hermaphrodite female parents using male sterility. **(B)** SI based systems as alternatives to androsterility for producing F1 hybrids. Self-(in)compatible parents and F1 hybrids are indicated. **(C)** Introgression of SI factors from different gene pools to restore SI in Brassicaceae SC lines. **(D)** Increase in F_1_ hybrid production by restricting SI alleles in semi-hybrid systems. References: [1] Kempe and Gils (2011); [2] Goring et al. (1992); [3] Rahman (2005); [4] Nasrallah et al. (2002); [5] Lin et al. (2015); [6] Riday and Krohn (2010); [7] Pembleton et al. (2015).

Hybrid vigor, or heterosis, occurs when two parents with different genetic backgrounds (usually pure lines) are crossed. Heterotic F_1_ progeny show elevated yield as well as other agriculturally desirable traits, such as enhanced resistance to abiotic stresses. The phenomenon is widespread and, consequently, hybrid cultivars are common in many crop species, including maize, sorghum, tomato, and pepper (Kempe and Gils, 2011). Since most cultivated crops are SC, producing hybrid seed requires an efficient system to control pollination to prevent the female parent from self-fertilization. Control methods range from mechanical emasculation and chemical gametocide agents to nuclear or cytoplasmic-encoded male sterility (with fertility restoration in the F_1_ hybrid) (Kempe and Gils, 2011) (**Figure 2A**). SI has been reported to be preferred over male sterility in crop species with entomophilous pollination since pollen-collecting bees rarely visit male-sterile plants (Kaothien-Nakayama et al., 2010). Nonetheless, SI also may have disadvantages, for example, F_1_ hybrids of two SI parents are also SI and this may be undesirable for crop production. SI F_1_ hybrids are not a handicap for ornamental or vegetable crops, but it may hinder seed production (e.g., oilseed rape/canola) or fruit production (e.g., stone and pome) (**Figure 2B**). Consequently, breeding programs favor not only SI female lines, but also SC F_1_ hybrids (Kaothien-Nakayama et al., 2010).

#### SI in Hybrid Breeding Schemes

In Brassicaceae, SI is widely used for hybrid seed production in the generally SI diploid vegetables *Brassica oleracea* and *B. rapa/B. campestris*. However, the derived amphi-diploid oilseed rape/canola (*B. napus*) is naturally SC, and introgression of *S*-alleles from its parental species is required to produce hybrid seeds. Thus, Goring et al. (1992) introgressed the *S*-locus from the SI *B. campestris* ‘W1’ line into the SC *B. napus* cv. ‘Westar’ and developed an SI ‘Westar’ line by backcrossing. Later, Rahman (2005) resynthesized SI in *B. napus* by crossing SI *B. oleraceae* (cv. ‘Green Duke’) and *B. rapa* (cv. ‘Horizon’, ‘Colt,’ and ‘AC Parkland’) (**Figure 2C**). Success in restoring SI across species led to hopes that a deeper understanding of the molecular genetics of SI might allow it to be more used extensively in hybrid breeding. Transfer of cloned *S*-genes from SI *Arabidopsis lyrata* to its SC relative *A. thaliana* has resulted in appropriate pollen rejection (Nasrallah et al., 2002) (**Figure 2C**), but transfer into distantly related crop species has yet to be reported. Indeed, it is unlikely to be successful since *Brassica* SI requires modifier genes for proper function as explained in *Molecular Mechanisms of SI*. However, *Papaver S*-genes may allow specific pollen rejection in a wide variety of species. Unlike the S-RNase-based systems and the *Brassica* SSI system, the *Papaver S*-specificity determinants (PrpS and PrsS) are sufficient for SI function. Lin et al. (2015) reported a proof-of-principle experiment in which *PrpS* and *PrsS* genes from *P. rhoeas* were transferred to *A. thaliana* (**Figure 2C**). The results showed a specific rejection response in *A. thaliana* that resembled *P. rhoeas* SI pollen rejection. As *P. rhoeas* and *A. thaliana* are distantly related, these results are very promising. Engineered crop incompatibility using *Papaver* SI genes may soon become a reality and this is the subject of a patent discussed further below.

SI is also promising for developing hybrid breeding systems in grasses. For instance, wheat (*Triticum aestivum* L.) is a fully SC inbred species where hybrid breeding to exploit heterosis has been hindered, in part, by the difficulty of using male-sterility. However, SI is present in other grasses such as rye. Thus, it might be possible to introgress genes from close SI relatives to generate SI wheat (Whitford et al., 2013). All studied grasses exhibit a well-documented gametophytic SI system controlled by two multiallelic loci, *S* and *Z*. In perennial ryegrass (*Lolium perenne* L.) two genes encoding DUF247 domain proteins of unknown function co-segregate with the *Z*- (Shinozuka et al., 2010) and *S*-loci (Manzanares et al., 2016) and have been proposed as candidate *S*-genes. This fine-scale mapping of the *L. perenne S*- and *Z*-loci will facilitate the identification of haplotype-specific markers allowing cross compatibility to be predicted. Thus, selection based on *S*- and *Z*-loci may be helpful for conventional ryegrass breeding where the inability to generate higher proportions of F_1_ hybrids has limited genetic gains associated with heterosis. For instance, in standard semi-hybrid systems, two heterotic populations (synthetic varieties) are allowed to interpollinate at the final phase of seed production, generally resulting in 50% hybrid seed. *S*-genotyping may improve this result. Simulations show that using two parental populations with restricted *S*-allele diversity (using linked markers for selection) may increase cross compatibility and generate up to 83.3% F_1_ hybrids (Pembleton et al., 2015) (**Figure 2D**). As a proof of concept, restriction of *S*-alleles in parent populations of the forage legume (Fabaceae) red clover (*Trifolium pratense* L.) allowed increased hybridity among seed produced. In controlled experiments, up to five populations containing just three *S*-alleles were randomly mated and compared to an *S*-allele unrestricted population. Only 48% of the seed from the unrestricted population was hybrid compared with 75% hybrids recovered from the restricted populations (Riday and Krohn, 2010) (**Figure 2D**).

#### Ways of Achieving SC and Its Role in Hybrid Breeding

Maintaining “pure” (highly homozygous) lines is also essential for hybrid breeding. Since SI species inherently favor outcrossing, achieving homozygosity may be hindered by the intra-specific crossing barrier itself and inbreeding depression. Maintaining SI parent lines with consistent properties may also be problematic. Consequently, breeders have used or considered a range of techniques to bypass SI including mentor pollen, bud and stump pollination, pollination of senescent flowers, irradiation, and high-temperature stress (De Nettancourt, 2001). Chemical treatments have also been employed. For example, treatment of pistils with NaCl or CO_2_ suppresses SI in SSI plants (Kučera et al., 2006). *In vitro* studies show that mixture of divalent zinc and copper ions inhibit S-RNases so it is conceivable this could suppress SI (Kim et al., 2001) (**Figure 1A**). More recently, a so-called “*in vitro* nursery” system has been suggested for SI crops. In this speculative scheme, gametes generated from somatic cells might be fused (i.e., simulating self-pollination), to obtain homozygous lines that could subsequently be used to produce single cross hybrids (De La Fuente et al., 2013) (**Figure 1A**).

Sources of SC for hybrid breeding may also be derived from spontaneous or induced mutations associated with loss of SI function in pollen, stigmas, or styles (see also *Different Sources of SC: A Favorable Trait for Yield Enhancement*). An illustrative example is the use of the SC-inducing *Sli* (*S*-locus inhibitor) gene in diploid potato. *Sli* was discovered in a SC variant of the wild potato relative *S. chacoense* and mapped to the distal end of chromosome 12 (Hosaka and Hanneman, 1998a; Hosaka and Hanneman, 1998b). Although not yet characterized at the molecular level, the *Sli* gene has been shown to act as a single dominant pollen factor that causes sporophytic inhibition of GSI (Hosaka and Hanneman, 1998a). In addition to having potential implications for understanding the GSI mechanism (McClure et al., 2011), *Sli* was soon perceived as a useful “tool” for selfing diploid potato to develop highly homozygous and seed-propagated lines (Phumichai et al., 2005). Thus, Jansky et al. (2014) developed M6, a vigorous diploid SC *S. chacoense* line that displays 90% homozygosity (including *Sli*). M6 is fully fertile, produces seeds when crossed with cultivated or wild potatoes, and will enable systematic creation of inbred diploid lines. However, the introgression of the *S. chacoense Sli* gene into other potatoes is time-consuming and may carry along undesirable traits such as long stolons or high tuber glycoalkaloid content. CRISPR knock-out of the *S-RNase* gene has recently been shown to be another viable method to generate SC potato lines avoiding linkage drag of undesirable traits associated with *Sli* (Ye et al., 2018; Enciso-Rodriguez et al., 2019). This important milestone has implications for the replacement of the current tetraploid asexually propagated potato with a diploid inbred line-based crop propagated by seeds (Jansky et al., 2016).

In perennial grasses, SI is often used in synthetic and hybrid breeding schemes. However, SC provides advantages to breeders including uniformity and propagation of parental inbreds and reduction of genetic load. Several routes to SC have been documented in grasses, including *S*- and *Z*-loci mutations (mainly pollen part defects) as well as other loci such as the *T*- (Thorogood et al., 2005) and the *SF*-loci (Do Canto et al., 2018). In practice, SC can be introgressed into populations of allogamous grasses by backcrossing. The derived inbred lines can be selected for heterozygosity at the *S*-locus using molecular markers, which would allow restoration of SI in the final synthetic varieties (Do Canto et al., 2016).

SC has been introduced in *Brassica* following both fairly traditional breeding methods as well as by directly manipulating *S*-gene expression (see *Different Sources of SC: A Favorable Trait for Yield Enhancement*). In one example, marker assisted selection (MAS) has been used to move two SC QTLs into SI cabbage (*B. oleraceae*) from line 87-534 (Xiao et al., 2019). One QTL, *qSC7.2*, was tightly linked with the *S*-locus, but the other, *qSC9.1*, was not associated to a known SI-related gene. Thus, although further research is needed to identify new SC genes, existing markers are already useful for MAS of SC (Xiao et al., 2019). Tantikanjana and Nasrallah (2015) proposed an alternative inducible SC system. It was mentioned earlier that expression of functional *S*-genes from *Arabidopsis lyrata* in SC *A. thaliana* pollen and stigmas causes SI. However, Tantikanjana and Nasrallah (2015) discovered that co-expression of both the *A. lyrata SRK* and *SCR* genes in *A. thaliana* stigma epidermal cells leads to ligand-mediated *cis*-inhibition of SRK and, thus, disrupts SI. This led them to propose two *cis*-SCR-based strategies for hybrid-seed production. Both strategies use a heat inducible promoter to allow inducible SC in otherwise SI inbreds (i.e., for maintenance) and in F_1_ hybrids for large-scale seed production.

### Overcoming Interspecific Reproductive Barriers (IRBs)

Interspecific hybridization is of great interest to plant breeders because valuable agronomic traits could, in principle, be captured by crossing with crop wild relatives. Wild genetic resources may find a variety of applications such as sources of biotic and abiotic stress resistances for use in elite cultivars. Other examples include generating interspecific hybrids for use as rootstocks and developing introgression lines for QTL mapping (Haussmann et al., 2004).

Accessing wild crop relative germplasm often first requires overcoming interspecific reproductive barriers (IRBs). Breeders have employed strategies similar to those used to mitigate intra-specific barriers: radiation-induced mutagenesis, mentor pollen, bud-pollination, temperature stress, and style removal (De Nettancourt, 2001; Van Tuyl and De Jeu, 2005). While these strategies have often succeeded, IRBs still represent an impediment to introgressing desired traits from wild relatives into certain crop-genera, including *Solanum* (Jansky, 2006; Bedinger et al., 2011), *Prunus* (Liu et al., 2007), *Cucumis* (Matsumoto and Miyagi, 2012), and *Fagopyrum* (buckwheat) (Mendler-Drienyovszki et al., 2013).

IRBs have been discussed in terms of two broad concepts, incompatibility and incongruity, that are best understood as distinct phenomena. Incompatibility describes pollinations that fail because an induced or constitutive pollen rejection mechanism. On the other hand, pollination can also fail because of divergent evolution and we regard this as incongruity. In an extreme example, wind-blown grass pollen may well land on a wet Solanaceae-type stigma, but this is not a congenial environment for germination and growth. These concepts are very helpful for understanding IRBs, but it is naïve to regard them as mutually exclusive.

Studies of interspecific unilateral incompatibility (UI) have helped elucidate the relationships between IRBs and SI. Since UI has been described in crop-families including Solanaceae, Poaceae, Brassicaceae, etc. (De Nettancourt, 2001), elucidating the mechanisms could be helpful to breeders. UI is defined as a form of incompatibility where crosses are successful in one direction, but not the other. Compatibility in one direction suggests that the parents are not so divergent that incongruity determines pollination behavior, so the failure of the opposite cross should be understood as incompatibility (Hancock et al., 2003). Often, UI follows the SI×SC rule (Lewis and Crowe, 1958) where SI species’ pistils reject pollen from SC species, but the reciprocal crosses are compatible. Although SI×SC UI relationships are common, UI can also occur in SC×SC or SI×SI interspecific crosses, but the latter are likely to be mechanistically distinct.

The first steps toward elucidating the mechanisms of UI have been taken in *Nicotiana* and *Solanum*. UI conforming to the SI×SC rule is very common in these genera suggesting that SI and UI are linked. Murfett et al. (1996) tested this hypothesis and confirmed that *Nicotiana* S-RNases are involved in some types of UI. Thus, S-RNases were the first factors identified as playing a role in pistil-side UI function, reinforcing a connection between SI and UI. However, the results also showed a requirement for additional (i.e., non-S-RNase) pistil-side factors in some types of UI (Murfett et al., 1996; McClure et al., 2000).

**Figure 3:**
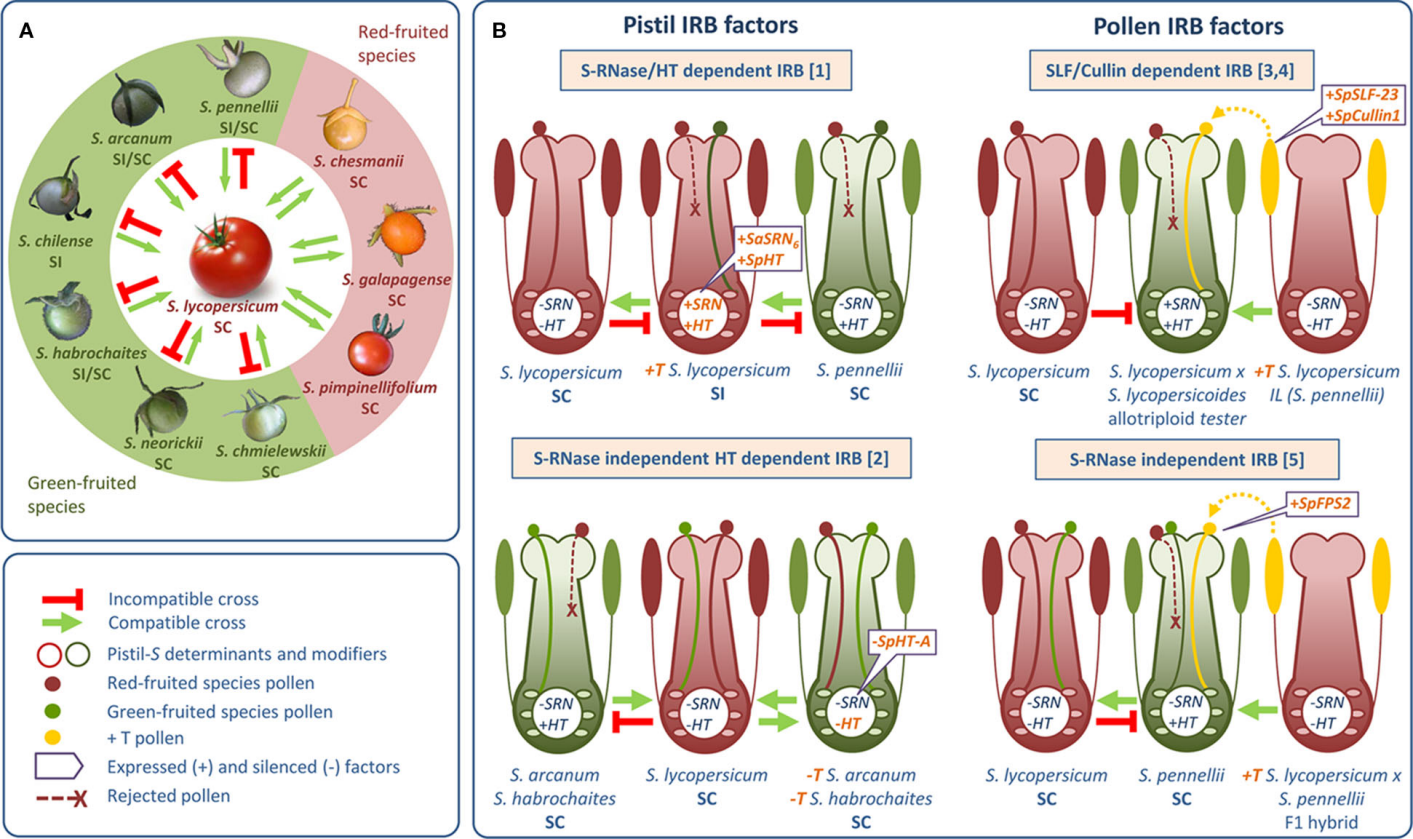
Interspecific Reproductive Barriers (IRBs) in *Solanum*. **(A)** Compatibility and incompatibility between *S. lycopersicum* and red- and green-fruited wild *Solanum* species. Direction of compatible and incompatible crosses is indicated by green *arrowed* and red *lines*, respectively. **(B)** Modification of IRBs in *Solanum* by introducing (+T) and/or knocking out (-T) pistil and pollen factors. Each modified IRB is indicated and referred: [1]Tovar-Méndez et al., 2014; [2] Tovar-Méndez et al., 2017; [3] Li and Chetelat, 2010; [4] Li and Chetelat, 2015; [5] Qin et al., 2018. Pollen and pistil transgenes are *orange-colored*. SC, self-compatible; SI, self-incompatible; *FPS*2, *Farnesyl pyrophosphatase synthase* gene; *SRN*, *S-RNase gene*; *SLF*, *S-locus F-box gene*; *HT*, *HT gene*; *Sp*, *Solanum pennellii*; *Sa*, *Solanum arcanum*.

Genetic studies implicated QTL on *Solanum* chromosomes 1, 3, and 12 in pistil-side UI function (Bernacchi and Tanksley, 1997), and additional evidence suggested that the chromosome 1 and 12 factors might correspond to *S-RNase* and *HT*-genes, respectively (Covey et al., 2010). Tovar-Méndez et al. (2014) tested this hypothesis by introducing functional *S-RNase* and *HT*-genes into cultivated tomato (*S. lycopersicum*) and reported that expressing both genes created a pollination barrier similar to the natural IRB separating the SC red/orange-fruited clade from the predominantly SI green-fruited species (**Figures 3A, B**). Neither *S-RNase* nor *HT* alone was sufficient to create the UI barrier. Thus, it is very clear that pistil-expressed SI genes contribute to some types of interspecific UI. However, it is also clear that other UI mechanisms also contribute. For example, some SC accessions lacking *S-RNase* still display UI (i.e., UI exists in certain SC×SC crosses). Intriguingly, HT-dependent S-RNase independent IRBs also have been reported in *Solanum* (Tovar-Méndez et al., 2017) (**Figure 3B**).

Pollen-side factors are also shared between SI and interspecific UI. Chetelat and DeVerna (1991) found genetic evidence for multiple UI factors and described major QTL controlling pollen-side UI expression on *S. pennellii* chromosomes 1, 6, and 10. Li et al. (2010) subsequently fine-mapped the *ui6.1* locus and identified the underlying gene as a pollen-expressed cullin gene (*CUL1*) that functions in both SI and interspecific UI (Li and Chetelat, 2010; Li and Chetelat, 2014). Later, the *ui1.1* locus was associated with a particular *S. pennellii SLF* gene, Sp*SLF-23*, further strengthening the connection between SI and UI (Li and Chetelat, 2015) (**Figure 3B**). Notwithstanding this relationship, Qin et al. (2018) recently identified an SI independent UI barrier in *S. pennellii* regulated by the pollen-expressed farnesyl pyrophosphatase synthase gene *FPS2* (**Figure 3B**).

Knowledge about mechanisms controlling interspecific barriers remains limited. However, in *Nicotiana* and *Solanum*, interspecific barriers have been directly manipulated (Murfett et al., 1996; Li and Chetelat, 2010; Tovar-Méndez et al., 2014; Tovar-Méndez et al., 2017) (**Figure 3B**). The *Solanum* model provides an excellent example. These studies show that UI mechanisms are *S*-locus dependent; silencing or overexpressing *S-RNases* could be useful for modifying both SI/SC phenotypes as well as UI. SI and UI are also likely to be related in other crop families, so it is possible that the *Solanum* results can be extended. For example, although more evidence is needed, preliminary results could suggest linkage between SI and UI in *Prunus* (Rosaceae), so this genus may be amenable to manipulation (Morimoto et al., 2019).

### Patented Applications of Plant SI Systems

The increasing number of SI-related patents attests to the commercial interest in practical application of SI. Data in **Table 2** were obtained from the PatentScope database (World Intellectual Property Organization) and list some of the most relevant SI-related patents since 2009, though the earliest SI-related patents date from the mid-1980s. Many of these were awarded to public institutions and universities, but private interests in the United States of America, China, and European Union are also represented. The patents are mainly focused on transgenic manipulation of SI and make claims including SI suppression, F_1_ hybrid production, overcoming interspecific barriers, and *S*-genotyping. Patents are based on SI factors from Papaveraceae, Brassicaceae, and Solanaceae, but also on the manipulation of SI in less studied families such as Poaceae and Orchidaceae.

**Table 2:** Patent applications related to self-incompatibility^a^.

Pub. n°	Title	Major claims	Potential uses	Crop species	Applicant (country)	Reference
CN 109750061	**Method for overcoming diploid potato self-incompatibility**	Development of diploid SC potatoes by knocking-out *S*-RNases usingCRISPR-Cas9 gene editing	Breakdown of SI Development of SC diploid lines for potato breeding	Potato	Agricultural Genomics Institute. Academy of Agricultural Sciences (China)	Sanwen et al., 2019
CN 106258956	**Pedigree breeding and wild transplanting method of *Dendrobium officinale***	Treatment of pistils with apollination accelerator (based on Indole Acetic Acid) to disturb SI	Improve the success rate and setting percentage of self-pollination	*Dendrobium officinale*	Sichuan Qiancao Biotechnology Co., Ltd. (China)	Xin et al., 2017
WO/2016/137029	**Primer set for assessing combination purity or discriminating genotype of cabbage class-II SI factor**	Class-II SRK genotyping by specific PCR amplification	Assess purity and discriminate genotypes to enhance hybrid seed production efficiency	Cabbage	Industry-Academic Cooperation Foundation of Sunchon National University (Korea)	Kang et al., 2016
US 2015/0322445	**SI system for making Brassicaceae hybrids**	Co-expression of Lal2and SCRL(*SRK* and *SCR* putative orthologs in *Leavenworthia alabamica*) polypeptides for conferring SI	Restoring of SI Obtaining of F_1_ hybrids for producing industrial grade oil	Brassicaceae (*Camelina sativa*)	The Royal Institution for the Advancement of Learning/McGill Univ. (Canada)	Schoen and Chantha, 2015
WO/2014/127414	**Manipulation of SI in plants**	A method for controlling hybridization *S* and *Z* haplotype candidate gene isolation Kit for controlling SI	Production of F_1_ hybrids Breakdown of SI	Poaceae (ryegrass [*Lolium* spp.] and fescue [*Festuca* spp.])	Agriculture Victoria Services Pty Ltd (Australia)	Spagenberg et al., 2014
WO/2014/115680	**Method for breeding *Brassica rapa* plant having SC**	A method to inactivate pollen-*S* factor SP11 while maintaining the inverted repeat sequence (SMI) on a class-I dominant *S*-haplotype	Breakdown of SI Production of F_1_ hybrids High seed-producing hybrids for edible or biodiesel purposes	Brassicaceae	National University Corporation Nara Institute of Science and Technology (Japan)	Takayama and Uno, 2014
CN 103710316	***Solanum chilense* SCF complex CUL1 subunit protein sequence and nucleotide sequence**	*CUL1* over- and/orunder-expression by genetic engineering	Overcoming incompatibility of distant hybridization with wild tomatoes Further research of SI mechanism	Solanaceae	Shanghai Jiao Tong University (China)	Zhao et al., 2014
WO/2014/029861	**Z locus SI alleles in Poaceae**	Identification of two glycerol kinase-like linked genes, LpGK1 and LpGK2, encoded by the *Z* SI locusin perennial ryegrass	Breakdown of SI Production of F_1_ hybrids Genotyping of *Z*-alleles Search for *Z* locus orthologs in Poaceae spp.	Poaceae	Aarhus Universitet (Denmark)	Studer and Asp, 2014
1020120001465	**RNA interference cassette for SI factor of *Brassica* spp. and a vector containing the same**	A vector containing apromoter, RNAi cassette with aSP11 pollen-*S* factor antisense from *Brassica rapa*, GUS^b^ region, and SP11 sense	Breakdown of SI	Brassicaceae	Industry-Academy Coop. Corps of Sunchon National University (Korea)	Shin et al., 2012
CN 102234324	**Protein involving SI and cross-compatibility control of phanerogam pollen, coding gene thereof, and application**	A vector containing a promoterand an RNAi cassette with a PhSSK1 pollen factor antisense from *Petunia hybrida*	Breakdown of SI	Solanaceae	Institute of Genetics and Developmental Biology, Chinese Academy of Sciences (China)	Zhao et al., 2011
WO/2010/061181	**Engineering of plants to exhibit SI**	Use of multi-allelic pollen- (*PrpS*) and pistil-expressed (*PrsS*)genes of the common field poppy (*Papaver rhoeas*) to confer SI on plants which do not possess a SI system	Transfer of SI into SCplants Production of F_1_ hybrids Prolong ‘shelf-life’ in ornamental plants and cut flowers	Papaver	The University of Birmingham (United Kingdom)	Franklin-Tong et al., 2010
1020090053403	**Primer set detecting SLG and SRK genotypes of radish SI**	A primer set fordetecting SLG genotype in radish. A PCR method using the primer set determine radish the genotype identity	Detect SI genotype of radish to prevent the failure of pollination and hybridization between radishes having the same SI genotype	Radish	Republic of Korea (management: rural development administration) (Korea)	Kim K. T. et al., 2009

a Data retrieved from the Patentscope database (WIPO, World Intellectual Property Organization), both national and international patent collections.

b GUS (β-glucuronidase reporter gene).

The relatively recent discovery that elements of the *Papaver* SI system may be used to transfer SI to unrelated species is especially noteworthy. The identity of the *S*-determinants and the physiological SI mechanism are briefly described in *Molecular Mechanisms of SI* and elsewhere (Wilkins et al., 2014). The dramatic demonstration that appropriate expression of *PrsS* and *PrpS* can confer SI on otherwise SC *A. thaliana* (De Graaf et al., 2012; Lin et al., 2015) formed the basis for the patent titled “Engineering of plants to exhibit self-incompatibility” by Franklin-Tong et al. (2010). This was not the first successful transfer of SI genes into *A. thaliana*, as Nasrallah et al. (2002) restored SI in *A. thaliana* by introducing SI genes from *A. lyrata*. However, the successful transfer of SI over the enormous phylogenetic distance between *Papaver* and *Arabidopsis* suggests that the *Papaver* system may find wide application. This *Papaver* based technology may offer advantages for crop breeding and production. Examples of potential applications include use as an alternative to male sterility for F_1_ hybrid production in species such as maize and rice, or as a means to avoid pollination induced senescence, thereby enhancing “shelf-life” of ornamentals or cut flowers, or preventing seed set in biomass crops. The *Papaver* SI-PCD system has also been envisioned as a potential research tool. For instance, McClure (2011) noted that *PrpS* genes might be used in cell fate studies to test the effects of removing specific cell populations by expressing the genes with cell-type-specific promoters and treating with the appropriate incompatible PrsS protein.

As noted, the grass GSI system is controlled by two multiallelic loci, *S-* and *Z-*, but the genes have not been positively identified. Nevertheless, two different patents have claimed the identification and use of *S-* and *Z-* candidate genes responsible for SI. Studer and Asp (2014) reported two glycerol kinase-like linked genes (*LpGK1* and *LpGK2*) as *Z*-locus candidates in perennial ryegrass. *Z*-allele specific variable regions in these genes allow prediction of *Z*-locus incompatibility phenotype and will be useful to control pollination in hybrid breeding systems. Spagenberg et al. (2014) reported the identification of a collection of genes located at the *S*- and *Z*-loci in perennial ryegrass and propose that modification (by gene editing) or selection (by using linked markers) of these genes might be useful for controlling hybridization.

Patents also have been issued taking advantage of the Brassicaceae and Solanaceae SI factors. Some highlights are mentioned to illustrate the range of applications. For instance, Shin et al. (2012) patented a system to suppress SI by blocking expression of the Brassica *SP11* pollen-*S* factor using RNAi. Takayama and Uno (2014) patented a non-transgenic method to inactivate *SP11* in *Brassica rapa*. This method involves crossing plants with class II *S*-haplotypes and plants carrying a dominant class I *S*-haplotype, but lacking *SP11*. This could be utilized in hybrid production of edible or biodiesel *Brassica* crops. Working in the opposite direction, Schoen and Chantha (2015) patented a method to restore SI, by co-expressing putative *SRK* and *SCR* orthologs from *Leavenworthia alabamica* in SC *Camelina sativa* to obtain F_1_ hybrids for producing industrial oils. In Solanaceae, Zhao et al. (2011) patented a system for eliminating SI in *Petunia hybrida* by suppressing *SLF-interacting*-*SKP*1*-like* gene (*SSK*) expression with RNAi. Zhao et al. (2014) patented the *Solanum chilense CUL1* sequence for use as a tool to overcome hybridization barriers in wild tomatoes. Recently, Sanwen et al. (2019) patented a procedure to knockout potato *S-RNases* using CRISPR-Cas9 as a means to develop potato diploid SC lines. In the genotyping domain, Kim K. T. et al. (2009) developed a method for *S*-genotyping radish (*Raphanus sativus*) cultivars by PCR-amplifying *SLG* alleles and, similarly, in cabbage Kang et al. (2016) patented a primer set for genotyping the *SRK* gene to assess combination purity.

SI-related patents have also been issued in species with unknown SI genetics. For instance, *Dendrobium* species are of interest as ornamental and medicinal plants, but show a high rate of SI and the system has not been characterized (Niu et al., 2018). Thus, in *Dendrobium officinale* (Orchidaceae) a method has been patented for overcoming SI using a pollination accelerator based on indole acetic acid (Xin et al., 2017).

## Future Prospects

**Table 3** presents a partial list of potential applications across a variety of crops. As noted, understanding the molecular basis of SI has enabled targeted manipulations for breeding and crop production. However, much remains to be learned and deeper knowledge may facilitate further improvements or enlarge the scope of possible applications. For instance, full molecular dissection of known SI systems may provide additional gene targets for creating SC (i.e. *Glyoxylase I* in canola, *ParMDO* in apricot, etc.). Furthermore, studies of as yet uncharacterized SI systems may facilitate applications (e.g., *S*-genotyping enabled pollenizer selection, developing SC cultivars, or hybrid breeding) in crops like tea, cocoa, olive, etc.

**Table 3:** Potential applications of SI research in crop breeding and production.

Crop type	SI system^a^	SI/SC^b^	Potential applications of SI research	Refs.
**Cereals crops**				
Wheat (*Triticum aestivum*)	GSI (*S*- and *Z*-loci)^a^	SC	Introgression of SI from close relatives for developing hybrid seeds	Whitford et al. (2013)
**Oilseed crops**				
Oilseed rape—canola (*Brassica napus*)	SSI	SC	Identification of new targets for inducing SC and methods to propagate SI lines for hybrid breeding	Yang et al. (2018)
Yellow mustard (*Sinapis alba*)	SSI	SI	Development of SI and SC inbred lines to produce high yielding synthetic varieties	Zeng and Cheng (2014)
**Vegetable crops**				
Tomato (*Solanum lycopersicum*)	S-RNase based GSI Non-self recognition	SC	Introgression of crop wild relative traits into elite cultivars by overcoming IRBs depending on SI Development of ILs for genetic analysis	Tovar-Mendez et al. (2017)
Cabbage, broccoli, etc. (*Brassica oleracea*)	SSI	SI	Identification of new target genes conferring SC Development of SC lines for hybrid breeding	Xiao et al. (2019)
**Tuber and root crops**				
Potato (*Solanum* tuberosum)	S-RNase based GSI Non-self recognition	SI/SC	Development of new CRISPR-KO SC diploid lines for efficient inbred/F1 hybrid strategies	Ye et al. (2018); Enciso-Rodriguez et al. (2019)
Radish (*Raphanus sativus*)	SSI	SI	*S*-genotyping for selecting weak SI plants as male and maintainer lines for hybrid breeding	Wang et al. (2019)
**Fruit crops**				
Cherry, almond, apricot, and plum (*Prunus* spp.)	S-RNase based GSI Self recognition	SI/SC	Identification of new SC sources Development of new interspecific hybrids on the basis of possible relation between SI and IRBs	Muñoz-Sanz et al. (2017b); Morimoto et al. (2019)
Apple, pear, and loquat (Rosaceae subf. *Maloideae*)	S-RNase based GSI Non-self recognition	SI/SC	*S*-genotyping for identifying new pollenizers Development of new SC cultivars	Claessen et al. (2019); Sheick et al. (2018)
Orange, mandarin, lemon (*Citrus* spp.)	Unknown (GSI)^a^	SC	Introgression of SI to reinforce seedlessness in commercial cultivars	Li et al. (2015); Kakade et al. (2017)
Olive tree (*Olea europaea*)	Unknown (DSI)^a^	SI	*S*-genotyping for selecting pollenizers Development of SC cultivars	Saumitou-Laprade et al. (2017)
**Beverage crops**				
Robusta coffee (*Coffea canephora*)	S-RNase based GSI^a^	SI	Overcoming SI to increase productivity and to facilitate breeding and crossing with *C. arabica*	Asquini et al. (2011)
Tea (*Camellia sinensis*)	Unknown (LSI)^a^	SI	Overcoming SI to develop SC homozygous lines that facilitate classical breeding	Zhang et al. (2016)
Cocoa (*Theobroma cacao*)	Unknown (LSI)^a^	SI	Prediction of SI/SC genotypes Selection/development of high-yield SC plants	Lanaud et al. (2017)

a SI systems where genetic control is unknown and/or whereS-determinants have not yet been identified. SSI, Sporophytic SI; GSI, Gametophytic SI; DSI, sporophytic Diallelic SI; LSI, Late acting SI; LSI.

b Predominant expressed phenotype.

There are some notable areas where improved understanding may contribute. Advances in omics and genome editing technologies are increasing the pace of identification of new SI factors. Nevertheless, omics studies in some species (e.g., tea, citrus, etc.) are exploratory and research is still needed before practical applications can commence. Besides analyzing the role of individual genes one by one in SI networks, SI studies are increasingly adopting a more quantitative approach and also considering environmental influences, phenotypic plasticity, and epigenetics. Nevertheless, our current knowledge of SI-environment interactions at the molecular level is very sparse and it remains to be seen how it will be applied. In addition, the cell-cell interactions underlying pollen-pistil recognition are also of potential importance because of their parallels to other self/non-self discrimination processes.

### Using Omics and Genome Editing Technologies to Elucidate SI

Omics technologies, which monitor classes of molecules (e.g., transcripts, proteins, etc.) are particularly well suited to understanding biological systems (Rhee and Mutwil, 2014). Applied to SI, they have the potential to efficiently elucidate the complete network of factors required for SI.

The availability of sequenced and annotated genomes in a broad range of species and accessions (Michael and Jackson, 2013) offers new opportunities to study SI. For instance, genomic sequences enhance the discovery of polymorphisms for refining the genetic and physical maps needed for positional cloning of new SI factors that could then be targeted for breeding. Thus, a GBS (Genotyping By Sequencing) based genome-wide association study identified two loci, CH1 and CH4, involved in the cocoa late-acting SI (LSI) system (Lanaud et al., 2017). Both CH1 and CH4 were associated with gametic selection, but only CH4 was associated with the resulting floral abscission. An ortholog of the *A. thaliana GEX1* (Gamete Expressed) protein, that is involved in male and female gametophyte development, was significantly associated with fruit set in the cocoa CH4 genomic region. Stone fruits (*Prunus*) provide further examples. Two SI modifier genes encoding thioredoxin-family proteins that are probably orthologs have been identified using independent genomics-based mapping strategies in apricot (Muñoz-Sanz et al., 2017a) and sweet cherry (Ono et al., 2018). In addition to identifying SI factors, these studies also provided markers that can readily be used in crop breeding to predict SI/SC phenotypes.

Transcriptomics may provide functional insights for putative SI factors. RNA-seq analysis has emerged as the preferred technique for transcriptome studies (Wang et al., 2009) and it has played an important role in elucidating the non-self recognition GSI system in Solanaceae and Rosaceae. It was used in *Petunia* to identify all the pollen specificity *SLF* genes (Williams et al., 2014; Kubo et al., 2015 ), and up to 24 *S-*locus *F-box Brothers* (*SFBB*) genes were similarly identified in *Malus* × *domestica* (Pratas et al., 2018).

RNA-seq also has been applied to better understand the molecular basis of SI in tea (*Camellia sinensis* (L.) Kuntze). SI in tea is not fully characterized but self-pollen rejection has been detected at the ovary consistent with LSI systems. According to Zhang et al. (2016), transcriptome analysis of self/cross-pollinated styles permitted identification of a differentially expressed gene (DEG) highly homologous to *S-RNase*. However, in a similar study, Ma et al. (2018) did not find *S-RNase* homologs, but instead identified other DEGs (e.g., aG-type *LecRLK*). More recently, Seth et al. (2019) constructed coexpression networks to detect transcripts groups with correlated profiles and also identified DEGs in self/cross-pollinated pistils. Tissue-specific qRT-PCRs confirmed DEGs in stigma-style and ovary, and support an LSI system that initiates in the style and extends to the ovary with the putative involvement of *S-RNase*, *SRK* and *SKP* genes in self-pollinations. Further research is still needed to clarify genetic control of SI in tea and provide useful tools for breeding.

Contributions to understanding SI from proteomics have been the subject of several reviews (Sankaranarayanan et al., 2013; Fu and Yang, 2014). Here, we highlight recent findings that point to future trends. The first proteomics-like studies of SI were conducted before the term “proteome” was coined and proved decisive in the identification of pistil *S*-determinant proteins in *Brassica* (Nishio and Hinata, 1980) and *Nicotiana* (Bredemeijer and Blaas, 1981). Thirty years later, new techniques based on 2-dimensional differential in-gel electrophoresis with or without coupling to MALDI-MS (Matrix Assisted Laser Desorption Ionization-Mass Spectrometry), identified up/down-regulated proteins potentially involved in SI. For instance, analysis of canola stigma proteins revealed the association between α2-4 tubulin levels and the microtubule network following pollen responses (Samuel et al., 2011). Chalivendra et al. (2013) used a tag-based proteomics technique called iTRAQ (Isobaric Tag for Relative and Absolute Quantitation) to follow the expression dynamics of SI factors in pistil development and in UI crosses with *S. pennellii*. This study laid the groundwork for identifying potential SI and UI factors (Bedinger et al., 2017).

The analysis of protein-protein interactions (PPIs) in a cell/tissue type, has special relevance for SI. These studies can be conducted *in vivo* using the yeast two hybrid (Y2H) system, or *in vitro* using affinity purification and pull-down or co-immunoprecipitation (Co-IP) followed by MALDI-TOF-MS (Morsy et al., 2008). For instance, Co-IP/MS of pollen extracts from a transgenic plant over-expressing a GFP-S_2_-SLF1 fusion protein allowed identification of SCF^SLF^ complex components in *Petunia inflata* (Zhao et al., 2010) and SSK1 and CUL1 homologs were similarly identified in sweet cherry (Matsumoto et al., 2012). Pull-down assays in *Solanum chacoense* showed that the pollen eukaryotic translation elongation factor 1Alpha (eEF1A) interacts with *S*-RNase and actin, suggesting that S-RNase may affect the actin cytoskeleton in SI (Soulard et al., 2014). S-RNase-binding pollen proteins also have been detected using Y2H in sweet cherry and apple (an actin homolog and an ABCF transporter, respectively) (Matsumoto and Tao, 2012; Meng et al., 2014).

In canola, proteomics (including Y2H) has facilitated identification of proteins that are down regulated after SI pollinations. Allele-specific recognition of male/female *S*-determinants (SP11/SRK) triggers phosphorylation of the ARC1 E3-ligase that then targets multiple compatibility factors for ubiquitination and degradation. These compatibility factors include Exo70A1, a component of the exocyst complex (Samuel et al., 2009), Glyoxylase I (GLO1), a stigmatic factor that detoxifies methylglyoxal (Sankaranarayanan et al., 2015) and phospholipase D1 (PLD1) that produces the necessary phosphatidic acid for exocytosis (Scandola and Samuel, 2019). In another study, Yang et al. (2018) used iTRAQ to study salt-induced SC in canola and found that GLO1 accumulated both in compatible pollinations and in incompatible pollinations after salt solution treatment, and proposed that salt-stress induced GLO1 expression leads to SC response. Thus, proteomics-based identification of proteins associated with salt-induced SC may provide new potential genetic resources for breeding SC lines.

Genome editing will be increasingly helpful for functional testing of putative SI factors. For example, CRISPR/Cas9-generated knockout mutants have been used to test the roles of SLF and SSK1 proteins in SI *Petunia* (Sun and Kao, 2018; Sun et al., 2018) and to demonstrate that a farnesyl pyrophosphatase synthase gene (*FPS2*) functions in *S-RNase*-independent UI in *Solanum* (Qin et al., 2018). As reported above, CRISPR knockout of *S-RNase* has also been successfully used to obtain SC diploid potato lines for breeding purposes. Other CRISPR-based genome editing tools, such as gene targeting, have not yet been applied to SI, but are also promising.

### Environmental Influences on SI: A Quantitative Perspective

Genetic and evolutionary models of SI typically assume qualitative inheritance. However, a growing body of evidence suggests that SI behaves as a quantitative trait in many species, and may be modulated by environmental conditions (Levin, 2012). Thus, global warming and its expected influence on sexual plant reproduction may become increasingly important. Temperature stress has been shown to affect post-pollination-prezygotic processes (i.e., when pollen-pistil recognition takes place) at several levels including pollen germination and viability, pollen tube growth and dynamics, and ovule viability (Hedhly et al., 2009). For example, temperatures ranging from 32 to 60°C can circumvent SI in some genera, though the underlying mechanisms are poorly understood (De Nettancourt, 2001). In Brassicaceae, Yamamoto et al. (2019) recently showed that high temperatures produce *S*-haplotype-dependent stigmatic SI breakdown by disrupting SRK targeting to the plasma membrane. Thus, *S*-haplotypes producing stable levels of SRK protein at elevated temperature, could facilitate production in SI parental lines for F1 hybrid seed production. Understanding how SI responds to elevated temperature may also help to control crop reproduction under a warming environment.

While it is not easy to forecast the effects of human disturbances, including global warming, on plant mating systems, it is reasonable to anticipate negative impacts on pollinators and mate scarcity, factors that could lead to limitations on availability of outcross pollen (Eckert et al., 2010). Indeed, limited availability of bees is already affecting crop management for entomophilous species (Nazzi and Pennacchio, 2014). In this context, reproductive assurance of SC cultivars would be valuable (Eckert et al., 2010) (see also *Pollenizers and Orchard Management*). For example, field based studies of SC sunflower show that the absence of pollinators does not affect yield (Astiz et al., 2011).

Pseudo-Self-Compatibility (PSC) systems that display a “leaky” SI response with some level of selfing can be regarded as a plant adaptation to address this challenge. PSC is commonly reflected by production of small fruits with a few small seeds after self-pollination, or by weak SI in older flowers (delayed selfing) (Busch and Schoen, 2008). It has been reported in several SI crops, including almond (Fernández i Martí et al., 2011), sweet cherry (Cachi et al., 2014), pear (Claessen et al., 2019), olive (Saumitou-Laprade et al., 2017), and grasses (Do Canto et al., 2016). Delayed selfing, which is frequently referred to as a “best-of-both-worlds” strategy because it combines the advantages of out-crossing, when possible, and selfing, when needed, has also been reported in several crop families (e.g., Solanaceae, Brassicaceae or Fabaceae) (Goodwillie and Weber, 2017). PSC is quantitative in nature and may be conditioned by the environment. Consequently, it is a variable trait dependent on season and genotype, which limits its usefulness in hybrid breeding and production. However, Do Canto et al. (2016) compiled examples where PSC was useful for hybrid breeding in grasses and proposed its use in distinct schemes for synthetic varieties.

Genetic dissection of PSC may facilitate its practical use and provide tools for fine tuning SI. Although the molecular mechanisms underlying PSC are poorly understood, known causes include, among others, down-regulation of *S*-genes and regulation by modifiers (Busch and Schoen, 2008). Chen et al. (2018) described an example of a modifier gene modulating the SI response. They found that, in pear, phosphatidic acid released by phospholipase D (PLD) may, at least initially, mitigate the toxic effects of the S-RNases in the incompatible pollen tube and delay SI signaling that leads to pollen tube death. In grasses, Do Canto et al. (2016) reviewed evidence supporting that PSC is polygenic in nature and dependent on the environment, and reported correlations between PSC and both *S*/*Z*-unlinked loci as well as with *S*/*Z*-haplotypes.

Accumulating evidence supports epigenetic regulation contributing to phenotypic plasticity of SI. In *Brassica*, Shiba et al. (2006) reported that the dominance relationships between allelic pollen *S*-determinants are controlled by allele-specific DNA methylation of recessive alleles. Tarutani et al. (2010) showed that this is attributed to the presence of an inverted repeat in dominant *SP11*/*SCR* alleles that produce a 24-nucleotide small RNA homologous to promoter sequences in recessive alleles. Subsequent epigenetic methylation is restricted to the anther tapetum and, therefore, it is not inherited (Fujii and Takayama, 2018). Nasrallah et al. (2007) reported two epigenetic mechanisms that cause SC in *Arabidopsis thaliana-lyrata* and *Capsella rubella-grandiflora* interspecific hybrids by producing aberrant *SRK* transcripts and suppressing *SCR* expression, respectively. Unlike *S*-locus genes mutations that cause irreversible loss of SI, such epigenetic changes are reversible. In almond, Fernández i Martí et al. (2014) recently showed that methylation of cytosine residues in the 5’ upstream region of the *S_f_-RNase* gene leads to the loss of SI.

Epigenetic modifications of SI may therefore be seen as new tools for crop improvement, especially for clonally propagated crops like fruit trees where seed propagation is not used.

### SI and Other Self/Non-Self Discrimination Systems

At the mechanistic level, SI entails cell-cell interactions that discriminate between self/non-self pollen (Nasrallah, 2005). In this sense, SI has parallels with other cell-cell signaling processes like innate immunity and pathogen defense (Sanabria et al., 2008), wounding (Pastuglia et al., 2002), perception of insect feeding (Bonaventure, 2012), and graft-induced stress (Cookson et al., 2014).

There are high-level parallels between plant immunity and SI. For instance, both discriminate against undesirable cells or organisms, and both rely on highly variable receptor-ligand molecules. The recognition receptor is even notably similar in some cases. For example, the *Brassica* SRK proteins are *S*-domain receptor-like kinases, and similar proteins (*S*-domain RLKs) have been shown to be up-regulated in response to wounding and pathogen infection and function by recognizing pathogen-derived “non-self” ligands (Sanabria et al., 2008; Catanzariti et al., 2015). Moreover, the SCR structure is similar to that of defensin proteins involved in defense against microbial infections in both plants and animals, suggesting a distant evolutionary link between SI and innate immunity in Brassica (Sanabria et al., 2008). There are also remarkable parallels between GSI systems and pathogen recognition. For example, programmed cell death is induced in poppy SI and in the plant immune response and Hugot et al. (2002) suggested that S-RNases may have evolved from defense-related RNases.

Plant recognition of phytophagous insects triggers a set of responses to elicit herbivore tolerance and it is thought that discrimination between the feeding behavior of piercing-sucking and chewing insects is a decisive step. The underlying receptor-ligand interactions have not been fully described, but a lectin receptor kinase1 (LecRK1) in *N. attenuata* was identified as a receptor candidate for perception of chewing by *Manduca sexta* larvae (Gilardoni et al., 2011). LecRK1 signaling suppresses feeding induced accumulation of salicylic acid, which allows the induction of jasmonic acid-regulated defense responses. LecRK1 is homologous to the *Brassica* SRK proteins and, like SRKs, contains a predicted N-terminal extracellular region, a single transmembrane domain, and a cytoplasmic serine/threonine kinase domain. However, they show clear differences in the extracellular binding domains. Potential ligands for LecRK1 are still unknown, but their identification would provide critical information about the mechanisms plants perceive insect feeding (Bonaventure, 2012).

Grafting is commonly used in vegetable and fruit-crops production to favor vigor and stress adaptation. The root system efficiency in grafted plants is directly linked to the compatibility between rootstock and scion and this is highly genotype-dependent. Thus, self/non-self discrimination may also be important for grafting success. For example, Cookson et al. (2014) found a set of receptor kinases differentially expressed in heterografting compared to autografting in grapevine (*Vitis* spp.) and this suggests some degree of non-self recognition in the hetrografts. Interestingly, these grape receptor kinase genes included homologs of *S*-locus glycoprotein-like gene family. Nevertheless it is important to note that in spite of these general similarities Irisarri et al. (2019) found no correlation between GSI and graft compatibility in the analysis of an apricot (*Prunus armeniaca*) F_1_ segregating population.

Thus, perhaps molecular level similarities may be more related to the limited gene repertoire of plants and the physical constraints of signaling than any fundamental similarity between pollen-pistil recognition and other self/non-self recognition. Nevertheless, further studies of SI and other self/non-self discrimination traits might reveal unexpected connections and lead to new applications.

## Concluding Remarks

Plant reproduction is crucial for crop breeding and production and SI systems are target traits for modulating reproductive behavior. Recent advances in understanding SI have already allowed intra- and interspecific barriers to be overcome and facilitated development of new plant materials (cultivars, hybrids, IL populations, etc.). Moreover, manipulating SI has been helpful to efforts to improve other crop traits such as seedlessness and fruit set. Emerging approaches (omics and genome editing) are also providing additional powerful tools to dissect SI cell-cell recognition mechanisms. Future SI studies will further assist breeders addressing crop production challenges including improvements in plant genetic resources utilization and mitigating effects of global warming on crops.

## Author Contributions

CR conceived the original idea. JM-S, EZ, FC-G, BM, and CR contributed to the main conceptual ideas and proof outline. JM-S, BM, and CR wrote the manuscript with support from EZ and FC-G. All authors provided critical feedback and contributed to the final version of the manuscript.

## Funding

This work was supported by grants of the Ministerio de Economia y Competitividad del Gobierno de España (AGL2010-19018 and AGL2015-64625-C2-2-R) and the Instituto Nacional de Investigaciones Agrarias (RF2011-00020-C02-02). We also acknowledge support for publication fee by the CSIC Open Access Publication Support Initiative through its Unit of Information Resources for Research (URICI).

## Conflict of Interest

The authors declare that the research was conducted in the absence of any commercial or financial relationships that could be construed as a potential conflict of interest.

## Abbreviations

ARC1, Arm Repeat Containing 1 U-box type E3-ligase; *CUL1*, *Pollen-expressed Cullin1* gene; GSI, Gametophytic Self-Incompatibility; IRB, Interspecific Reproductive Barriers; LSI, Late acting Self-Incompatibility; MLPK, M-Locus Protein Kinase; ParMDO, *Prunus armeniaca* M-locus Disulfide Bond A-like Oxidoreductase; PCD, Programmed Cell Death; *SSK1*, SLF-interacting-SKP1-like gene; PSC, Pseudo-Self-Compatibility; RNAi, RNA interference; SC, Self-Compatibility/Self-Compatible; SCR/SP11, *S*locus Cysteine-Rich protein; SI, Self-Incompatibility/Self-Incompatible; SKP, Suppressor of Kinetochore Protein; SLF/SFB*, S*-Locus F-box proteins; SLG, *S*-Locus Glycoprotein; *Sli*, *S*-locus inhibitor gene; SRK*, S*-locus serine/threonine Receptor Kinase; SSI, Sporophytic Self-Incompatibility; UI, Unilateral Incompatibility.
